# Exploring the Safety and Tolerability of IGC‐AD1 in Alzheimer’s Patients: Insights from CYP2C9 Polymorphism Assessment

**DOI:** 10.1002/alz.095385

**Published:** 2025-01-09

**Authors:** Marian A Sulvaran, Maria Juanita Arbelaez, Evelyn Gutiérrez, Maria Alejandra Tangarife, Jagadeesh S Rao, Margarita Venegas, Laura Tatiana Sánchez, Saadia Shahnawaz, William Julio De Jesus, Karen Y Pujals, Claudia Grimaldi, Elliot Hong, Ram Mukunda

**Affiliations:** ^1^ IGC Pharma, Bogotá, Bogotá D.C. Colombia; ^2^ IGC Pharma, Potomac, MD USA; ^3^ Santa Cruz Behavioral, Bayamon, PR USA; ^4^ UTHealth Houston McGovern Medical School, Houston, TX USA

## Abstract

**Background:**

Alzheimer’s disease (AD) affects millions of Americans, with potential future increases without breakthroughs in treatment. IGC‐AD1, a novel formulation comprising of delta‐9 tetrahydrocannabinol (“THC”) and melatonin, is being studied in AD‐associated agitation. THC is predominantly metabolized by cytochrome P450 and specifically by CYP2C9. We present the comparison of Adverse Events (“AE”) between two polymorphisms of CYP2C9, intermediate (“IM”) and normal metabolizers (“NM”).

**Methods:**

Thirteen Puerto Rican AD patients participated in a three‐cohort MAD, Phase‐1 safety, and tolerability trial (IND146069, NCT04749563). In Cohorts‐1, 2, and 3, one mL of IGC‐AD1 was administered QD, BID, TID, respectively, for 14‐days, with a minimum washout period of 4‐days between cohorts. CYP2C9 genotyping was assessed. Active Participants were grouped by phenotypes, Intermediate, or Normal. The IM group was assessed by genotype (*1/*2, *1/*3). Frequency of Adverse Events (“AE”s) was evaluated by group. Chi‐squared was used to compare differences between groups (SPSSv.28).

**Results:**

Seven patients were IM (allele *1/*2 = 4, allele*1/*3 = 3), while five were NM. Both groups IM and NM presented somnolence (IM: 57%; NM: 80%, p = 0.63), hypertension (IM: 43%; NM: 20%, p = 0.50), dizziness (IM: 43%; NM: 40% p = 0.94), and falls (IM: 29%; NM: 40% p = 0.74). No serious adverse events were reported.

**Conclusion:**

In this limited sample size and trial, there were no significant differences in AE frequencies between the two CYP2C9 phenotypes IM and NM, suggesting that IGC‐AD1 has a safety profile that does not vary greatly, based on these two CYP2C9 polymorphisms. This analysis and initial results are important because the pharmacokinetics of THC are different for IM and NM. We continue to study these and other polymorphisms of CYP2C9 to understand their impact on AEs as it is crucial for personalized treatment strategies for the aged AD population.

